# Comparison Between Pantoprazole Intermittent Dosing and Continuous Infusion in Suspected Upper Gastrointestinal Bleeding Prior to Endoscopy: Impact of a Pharmacist-Driven Protocol to Reduce Utilization of Pantoprazole Continuous Infusion

**DOI:** 10.7759/cureus.48056

**Published:** 2023-10-31

**Authors:** Ivan A Hernandez, Jason Morell, Lauren Mulcahy, Daniela Luzardo

**Affiliations:** 1 Pharmacy, Baptist Health South Florida, Miami, USA; 2 Pharmacy, University of Florida, Miami, USA

**Keywords:** clinical pharmacist, gastroenterology and endoscopy, s: acute variceal upper gastrointestinal bleed, proton pump inhibitor, acute gastrointestinal bleed

## Abstract

Background: Current practice for patients with suspected or confirmed upper gastrointestinal bleeding (GIB) is to utilize a proton pump inhibitor (PPI) bolus followed by a continuous infusion for 72 hours. Literature has shown similar outcomes with intermittent bolus dosing compared to continuous infusion. Substitution would lead to reduced costs and utilization of resources.

Methods: This was a retrospective case-control study conducted via chart review. Utilizing electronic healthcare record reports, patients in the control arm were screened for inclusion if they received a pantoprazole continuous infusion from December 1, 2020, to March 31, 2021. A total of 38 patients were included in the control arm. Patients in the experimental arm were screened for inclusion with pantoprazole intermittent therapy from January 1, 2022, to June 30, 2022. A total of 60 patients were included in the experimental arm. The primary outcome was a 30-day GIB recurrence. Secondary outcomes included 30-day hospital readmission, 30-day *Clostridioides difficile* (*C. difficile*), hospital length of stay (LOS), and number of pantoprazole vials utilized.

Results: There was a 65% reduction in the 30-day GIB recurrence in the intermittent bolus arm compared to the continuous infusion arm. Thirty-day hospital readmission was 57% lower in the intermittent bolus arm compared to the continuous infusion arm. The LOS between the two arms was almost identical with the median being five days for the intermittent bolus arm and the median being four days for the continuous infusion arm. The 30-day *C. difficile* infection rate had 5% of patients acquiring *C. difficile* in the intermittent bolus arm and 2.5% in the continuous infusion arm. The intermittent bolus arm used 55% fewer pantoprazole vials than the continuous infusion arm.

Conclusion: In hospitalized patients, the utilization of pantoprazole intermittent bolus is not only comparably efficacious but potentially represents a safer and economically advantageous alternative compared to the current guideline recommendation of a 72-hour pantoprazole continuous infusion. Further studies could provide more robust data to support our findings and challenge the current recommendation for patients who meet the indication criteria.

## Introduction

Upper gastrointestinal bleeding (GIB) is associated with a mortality rate of 5-10% [1]. By decreasing the pH below a level of 6, pepsin evades its ability to lyse the aggregation of platelets and coagulation [2]. Compared to placebo, a proton pump inhibitor (PPI) bolus followed by continuous infusion maintained a pH below 6; thus, this regimen was historically utilized in patients with a GIB [3]. The 2010 International Consensus Recommendations on the Management of Patients with Nonvariceal Upper Gastrointestinal Bleeding (UGIB) recommends a PPI bolus followed by continuous infusion for 72 hours if high-risk stigmata bleeding [4]. In 2014, a meta-analysis published by Sachar et al. compared intermittent boluses with continuous infusions of PPI [5]. His practice-changing results challenged the guidelines, indicating no significant difference between the two arms. Subsequently, multiple meta-analyses have been conducted, consistently demonstrating that intermittent PPI dosing is non-inferior to continuous infusion PPI approach.

Several studies reviewed the safety risks associated with utilization of PPIs [6-9]. A retrospective cohort study conducted in two Quebec hospitals examined the risk of *Clostridioides difficile *(*C. difficile*)-associated diarrhea (CDAD) using continuous PPI therapy [10]. The results revealed that patients in this study were approximately 1.5 times more likely to have a CDAD infection if on continuous PPI therapy than if not on PPI therapy. Another observational trial studied the association between PPI use and community-acquired pneumonia. The authors found that the strongest association occurred within the first week of exposure to PPI therapy and persisted after 90 days of exposure [11]. PPIs have also been implicated with electrolyte imbalances, specifically associated with serum hypomagnesemia, which may cause cardiac abnormalities [12]. In light of these substantial adverse effects of long-term PPI utilization, hospital pharmacists can play a pivotal role in safeguarding patients from these potential complications. By exercising their expertise, hospital pharmacists can contribute significantly to the optimization of PPI treatment, ensuring that its benefits outweigh the potential harms.

The Forrest classification can help differentiate between the different types of upper stigmata GIBs. A Forrest I lesion is an actively bleeding ulcer, a Forrest IIB lesion is an ulcer with adherent clots, and a Forrest IIC lesion is an ulcer with red spots [13]. The different Forrest classifications can help determine how severe the bleeding is. The more severe the ulcer, the more likely the patient is to have re-bleeding. Forrest I, IIA, and IIB are more severe bleeding versus Forrest IIC and II which are low-risk ulcers.

In 2021, a critical shortage of pantoprazole vials emerged [12]. This shortage prompted a more strategic utilization and alignment of dispensing practices in accordance with evidence-based guidelines. The primary clinical indication for pantoprazole vials is for the management of UGIB for patients with gastroesophageal reflux disease (GERD) who cannot tolerate oral medications. At the study institution, a considerable number of patients who did not have a bleeding ulcer received pantoprazole continuous infusions. These patients received a pantoprazole infusion order upon admission due to a variety of factors such as an occult positive stool sample, instances where patients reported the ingestion of substances like bismuth subsalicylate that resulted in dark stools, and other non-GIB related incidences. Due to the evolving situation, in April 2021, a multidisciplinary plan was formed by pharmacists and gastroenterologists at the institution to systematically guide and steward the use of PPIs, with a focus on intravenous pantoprazole. As a result of this initiative, a stringent criterion was established to limit pantoprazole infusions to patients who met the following criteria: patients who had undergone an esophagogastroduodenoscopy with documentation of a high-stigmata upper GIB or patients who presented with hematemesis [14,15]. If the patient did not meet the criteria, then the continuous PPI was interchanged to a loading dose of a PPI bolus followed by intermittent boluses. The purpose of the investigation is to ensure stewardship of PPI therapy in the context of UGIB to achieve quality and safety outcomes.

## Materials and methods

Study design

A retrospective case-control study was conducted to examine the relationship between PPI continuous infusion and intermittent bolus therapy associated with a 30-day recurrence of GIB. The control arm consisted of 38 patients who received a pantoprazole continuous infusion due to a suspected GIB during hospitalization from December 1, 2020, to March 31, 2021, which was prior to the shortage. These patients were reviewed to ensure alignment with guideline recommendations for the appropriate indication and utilization of patients with bleeding ulcers with high stigmata upper GIB. Many patients did not meet the guideline recommendations for a continuous PPI infusion. Based on the findings gathered from the pantoprazole medication utilization review in our control arm, we implemented a protocol to restrict the eligibility for administration of pantoprazole continuous infusion to a certain criterion. Patients who did not meet this criterion were transitioned to an 80 mg IV push loading dose of pantoprazole followed by a pantoprazole 40 mg intermittent bolus twice-daily regimen. After the intervention, the experimental arm constituted 60 patients who received pantoprazole 40 mg intermittent bolus twice daily due to a possible GIB from January 1, 2022, to June 30, 2022. Data collection for both arms was achieved utilizing a report and manual chart review from the electronic health record. Investigational Review Board approval was waived.

Statistical analysis

Data analysis for the 30-day GIB recurrence, 30-day hospital readmission, and 30-day *C. difficile* were conducted employing the two-tailed Fisher exact probability test, and a p-value of 1 was calculated. The primary endpoint was reported as a percentage and secondary endpoints as follows: 30-day readmission as a percentage, 30-day incidence of *C. difficile* as a percentage, and LOS and number of pantoprazole vials as median from patient total. The investigators of the study conducted all data analyses.

Study endpoints

The primary endpoint was the evaluation of the 30-day GIB recurrence. The secondary endpoints included 30-day hospital readmission rates, 30-day incidence of *C. difficile*, as defined by the presence of both toxin and DNA in stool, hospital LOS, and the total number of pantoprazole vials consumed throughout the treatment.

## Results

A total of 98 patient electronic health records were reviewed from December 1, 2020, to June 30, 2022, with 38 patients in the control arm treated with PPI infusion and 60 patients in the experimental arm treated with PPI intermittent bolus.

Study population

The baseline patient demographics and characteristics for all 98 patients included in the study are outlined in Table 1. The mean age of the subjects across both study arms was 77 years old, with roughly 50% and 34% of the participants being male in the pantoprazole infusion arm and intermittent bolus arm, respectively. Among the 38 patients who received continuous PPI treatment, 18% presented with hematemesis, 54% with melena, 7.7% simultaneously exhibited both hematemesis and melena, and 21% presented with neither. Among the 60 patients treated with intermittent PPI therapy, 28% presented with hematemesis, 53% with melena, 6.7% simultaneously exhibited both hematemesis and melena, and 11.6% with neither. The average hemoglobin was consistent at 9.6 g/dL in both arms, and the average number of packed red blood cell (PRBC) units administered was 1.08 units in the pantoprazole infusion arm and 0.7 units in the pantoprazole intermittent bolus arm. In addition to the aforementioned parameters, various other patient factors were assessed, including current smoking status, alcohol consumption, aspirin and/or non-steroidal anti-inflammatory drug (NSAID) utilization, anticoagulation therapy history prior to admission, comorbidities encompassing renal, cardiac, respiratory, and other medical conditions, and the specific site of the GIB.

**Table 1:** Baseline patient characteristics EtOH: ethanol, NSAID: non-steroidal anti-inflammatory drug, GIB: gastrointestinal bleeding, PRBC: packed red blood cells, ICU: intensive care unit

Factors	Continuous infusion arm (n=38)	Intermittent bolus arm (n=60)
Age (avg)	77.9 (SD 12.07)	77.3 (SD 13.67)
Sex (% male)	51%	34%
Hemoglobin mg/dL (avg)	9.6 (SD 3.16)	9.6 (SD 3.08)
Clinical Presentation		
Hematemesis	7 (18%)	17 (28%)
Melena	21 (54%)	32 (53%)
Both	3 (8%)	4 (7%)
Neither	7 (21%)	7 (12%)
Smoker	16 (41%)	20 (33%)
EtOH use	14 (36%)	16 (27%)
Aspirin use	8 (21%)	11 (18%)
NSAID use	4 (10%)	8 (13%)
Anticoagulation prior to admission	13 (33%)	24 (40%)
Comorbidities		
Renal	11 (28%)	11 (18%)
Cardiac	24 (62%)	37 (62%)
Respiratory	11 (28%)	13 (22%)
Other	36 (92%)	45 (75%)
Location of bleeding		
Upper GIB, no varices	14	13
GIB suspected, never found	13	30
Lower GIB	10	15
Esophageal Varices	1	2
PRBC (avg)	1.08 (SD 1.6394)	0.7 (SD 0.97945)
ICU stay (# of patients)	15	7

Clinical endpoints

The primary endpoint of the study, which focused on the assessment of the 30-day GIB, along with secondary endpoints, such as the 30-day readmission rates, was the 30-day incidence of *C. difficile*.

**Figure 1:**
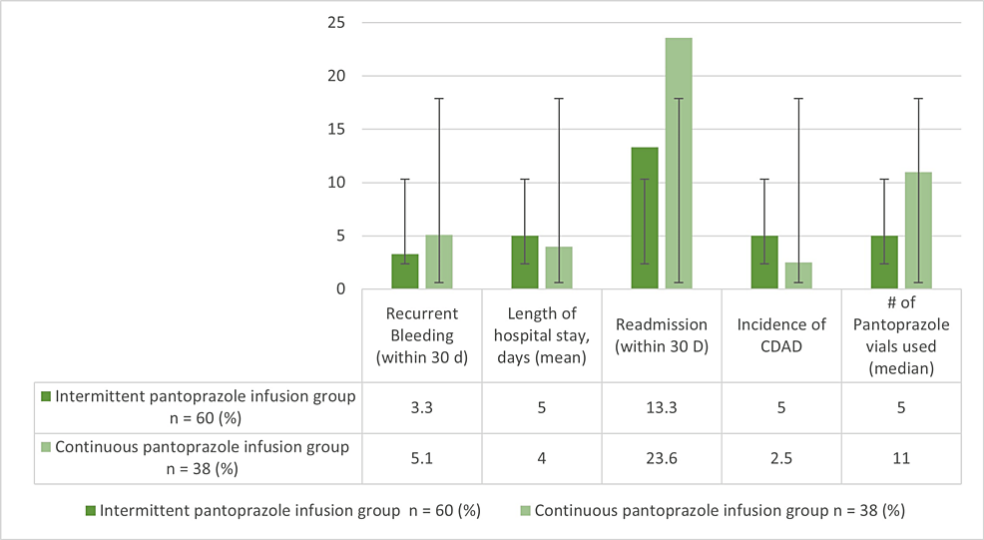
Primary and secondary endpoint results CDAD: *Clostridioides difficile*-associated diarrhea

**Figure 2:**
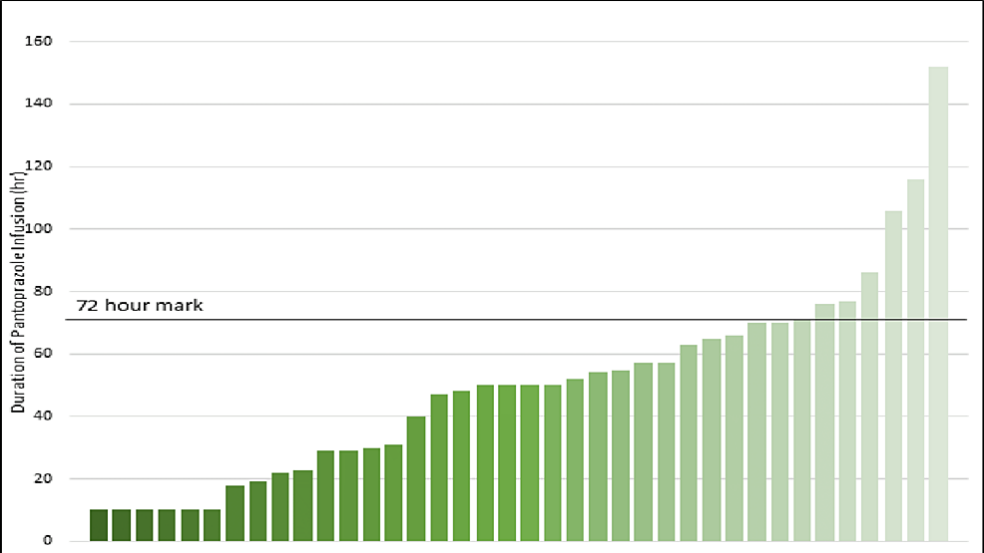
Duration of pantoprazole infusion in the control arm

The 30-day GIB recurrence was 7% in the control arm and 3% in the experimental arm. The 30-day hospital readmission was 33% in the control arm and 13% in the experimental arm. The 30-day *C. difficile* was 4% in the control arm and 5% in the experimental arm. As anticipated, there was a significant reduction in the utilization of pantoprazole vials in the intermittent bolus PPI treatment arm, with a median requirement of five vials, contrasting with the median consumption of 11 vials in the continuous infusion arm. The median LOS was lower in the continuous infusion arm by one day (Figure 1). Furthermore, an in-depth assessment of the patients who received pantoprazole via continuous infusion revealed instances where the infusion duration exceeded the guideline-directed recommendation of a maximum of 72 hours. In particular, 15% of patients exceeded this duration, with one receiving an infusion of approximately 150 hours, as illustrated in Figure 2.

## Discussion

This retrospective case-control study has shown that intermittent and pantoprazole continuous infusion therapies have comparable efficacy in patients with suspected upper GIB to the current guideline-directed regimen. With a calculated p-value of 1, the primary and secondary endpoints were not statistically significant. This is possibly due to the low sample size. This observational study was likely underpowered. The primary objective of this medication use evaluation is to improve patient outcomes and quality of life by assessing clinical outcomes via a multidisciplinary approach.

The American Society of Health Systems Pharmacists suggests the FOCUS-PDCA methodology for medication use evaluations, which was employed by the study institution [16]. The target for improvement was found while reviewing current pantoprazole infusion practices in light of a nationwide shortage. The data outlined in Figure 2 showcases the third step in the FOCUS-PDCA methodology of clarifying the current practice patterns.

In Figure 1, there is a comparison between the control and experimental arms for the 30-day GIB risk. The continuous infusion shows a higher GIB risk in 30 days. A similar trend is observed with secondary endpoints, which include 30-day hospital readmission, 30-day *C. difficile*, LOS, and amount of pantoprazole vials administered. Figure 2 shows that 15% of the patients in the control arm received a pantoprazole continuous infusion that exceeded the recommended duration of 72 hours, with one patient receiving an infusion that nearly doubled the recommended threshold.

Notably, the LOS in both arms exhibited minimal disparity, with a mere one-day difference between them. This observation may suggest that patients can be managed safely and effectively with a pantoprazole intermittent bolus regimen without extending their hospitalization.

The amount of pantoprazole vials used was substantially reduced by over 50% in the experimental arm. Given an average pantoprazole vial cost of $2.25 for the study institution at the time of this writing, the per-patient cost in the intermittent bolus arm amounted to $11.25 compared to $24.75 in the continuous infusion arm. This translates to a notable overall cost reduction of $13.50 per patient, favoring the intermittent pantoprazole bolus regimen.

Not only was there a reduction in cost between the two arms, but an increase in ease of use was noted for both the pharmacy and nursing departments. At the time of this study, pantoprazole infusions were being compounded in a sterile environment, sometimes leading to delays in administration. In contrast, the vials used for intermittent bolus therapy were readily available in an automated medication dispensing system. The continuous infusion at the study institution was replaced with a simple IVP twice daily. By reducing the need for frequent replacement and assessment, there was a potential enhancement in efficiency and workload management.

These results are comparable to previous studies in relation to showing no harm between using PPI continuous infusion and PPI intermittent boluses [6-9]. This study can be used in prior studies and meta-analyses to further advance the knowledge that PPI intermittent boluses can be used in GIB with no additional harm compared to PPI continuous infusions.

This study has limitations that could have influenced the results. Due to the small sample size, a p-value of 1 was calculated, making the study not statistically significant. Additionally, the study was not randomized. There was also no Forrest classification included in the electronic health record. Many institutions do not use Forrest classifications due to the decrease in *Helicobacter pylori* and the increase in the use of PPIs, causing fewer peptic ulcers. Another possible flaw with this study is that there were 38 patients in the control arm and 60 patients in the experimental arm. The reason the experimental arm is larger is that there were providers who believed PPI intermittent bolus was just as effective as continuous PPI during the control arm phase. Therefore, patients were getting intermittent bolus PPIs during the control arm period that were not captured. This would make the experimental arm larger since it captured the PPI boluses that were not interchanged and the ones that were. There were also confounders. Patients with lower GIBs and suspected GIBs that were never found were included in the control arm to justify the protocol of switching continuous PPI to intermittent boluses. Due to this, they were also included in the experimental arm for continuity. All these factors could have influenced the results.

Clinical practice would benefit from more robust studies with a larger sample size to validate our findings more conclusively. Another limitation was the disparity in patient distribution between the study arms. However, rigorous efforts were made to mitigate this discrepancy by stratifying patients into multiple subcategories and employing matching techniques to foster comparability to the greatest extent feasible.

## Conclusions

This retrospective case-control study demonstrated that the utilization of pantoprazole intermittent bolus is not only comparably efficacious but also potentially represents a safer and economically advantageous alternative to the current guideline approach of a 72-hour pantoprazole continuous infusion. While the findings from this study did not show statistical significance, we believe there is a clear clinical benefit. Further studies could provide more robust data to support our findings and challenge the current recommendation for patients who meet the indication criteria.
